# Radiation-Induced Senescence Bystander Effect: The Role of Exosomes

**DOI:** 10.3390/biology9080191

**Published:** 2020-07-27

**Authors:** Eman Elbakrawy, Savneet Kaur Bains, Scott Bright, Raheem AL-Abedi, Ammar Mayah, Edwin Goodwin, Munira Kadhim

**Affiliations:** 1 Department of Biological and Medical Sciences, Faculty of Health and Life Sciences, Oxford Brookes University, Gipsy Lane, Headington, Oxford OX3 0BP, UK; emanoxford2018@gmail.com (E.E.); savi.kaur.bains@hotmail.co.uk (S.K.B.); 14107920@brookes.ac.uk (R.A.-A.); amayah@brookes.ac.uk (A.M.); 2 Department of Radiation Physics, National Center for Radiation Research and Technology, Atomic Energy Authority, 3 Ahmed El-Zomor Al Manteqah Ath Thamenah, Nasr City, Cairo 11787, Egypt; 3 Department of Radiation Physics, University of Texas MD Anderson Cancer Centre, 1515 Holcombe Blvd, Houston, TX 77030, USA; SJBright@mdanderson.org; 4 Angelina Biomedical Laboratories, 2110 Deer Valley Lane, Laporte, CO 80535-9750, USA; Eds_email@msn.com

**Keywords:** senescence, exosomes, radiation, bystander, the role of exosomes

## Abstract

Ionizing Radiation (IR), especially at high doses, induces cellular senescence in exposed cultures. IR also induces “bystander effects” through signals released from irradiated cells, and these effects include many of the same outcomes observed following direct exposure. Here, we investigate if radiation can cause senescence through a bystander mechanism. Control cultures were exposed directly to 0, 0.1, 2, and 10 Gy. Unirradiated cells were treated with medium from irradiated cultures or with exosomes extracted from irradiated medium. The level of senescence was determined post-treatment (24 h, 15 days, 30 days, and 45 days) by β-galactosidase staining. Media from cultures exposed to all four doses, and exosomes from these cultures, induced significant senescence in recipient cultures. Senescence levels were initially low at the earliest timepoint, and peaked at 15 days, and then decreased with further passaging. These results demonstrate that senescence is inducible through a bystander mechanism. As with other bystander effects, bystander senescence was induced by a low radiation dose. However, unlike other bystander effects, cultures recovered from bystander senescence after repeated passaging. Bystander senescence may be a potentially significant effect of exposure to IR, and may have both beneficial and harmful effects in the context of radiotherapy.

## 1. Introduction

### 1.1. Ageing and Senescence

One major aspect of aging at the cellular level is senescence. Cellular senescence is triggered by various factors such as telomere attrition, enhanced oxidative stress, and accumulation of DNA damage [1]. Senescence has always been characterized as an arrest in cell growth, but the more it is investigated, the more it becomes apparent it is a dynamic process that also impacts on development and tissue repair [2]. Traditionally, senescence has been thought to occur in a similar manner independent of inducing factors; however, there is emerging evidence of differences based on the inducing agent, for example, epigenetic alterations and DNA damage responses (DDR) in response to ionizing radiation (IR) exposure or naturally induced senescence through the recognition of short telomeres [3]. Senescent cells also demonstrate a secretory phenotype called a senescence-associated secretory phenotype (SASP), which is important in relation to intracellular signaling/cell communication and tissue effects. It has been hypothesized that as more cells in a given tissue enter the senescent state, potentially driven by the SASP, tissue repair becomes increasingly inefficient, which may contribute to the general loss of organ functionality that characterizes aging [3]. Recent research has demonstrated that the SASP’s potent autocrine and paracrine activities induce inflammation and fibrosis, induce malignant phenotypes, and attract immune cells to senescent cells themselves and the neighboring cells, which change tissue microenvironments and lead to aging and age-related diseases [1,4,5,6]. Recent study showed that senescent cells secreted bioactive factors into the blood that changed hemostasis and drove blood clotting [7]. These SASP factors therefore could work as plasma biomarkers for aging and age-related diseases that are distanced by the existence of senescent cells [8].

### 1.2. IR and Senescence

IR is known to induce cell senescence in healthy cells and therefore has the potential to accelerate aging and the early onset of diseases associated with aging [9]. It is currently unclear if the underlying molecular mechanisms behind radiation-induced senescence (RIS) are the same as those that occur in senescence associated with normal aging. The molecular mechanisms involved in radiation-induced cellular responses depend on a number of factors: radiation dose, dose rate, transformed status, cell type, and growth rate [10].

### 1.3. Non-Targeted Effects of Radiation

IR is also known to induce non-targeted effects (NTE) [11,12,13]. Manifesting as typical radiation effects, such as DNA damage, NTE are temporally or spatially separated from the initial radiation exposure. Two of the more common examples of NTE are (i) genomic instability (GI), which occurs in the descendants of irradiated cells and typically is observed as the delayed appearance of new genetic or epigenetic alterations, and (ii) bystander effects (BE), which occur in cells that were not directly irradiated but have been allowed to exchange molecular signals with cells that were irradiated [12]. Bystander effects are mediated through gap junction communication and/or secreted signals (bystander signals) from neighboring irradiated cells [11,12]. The nature of secreted signals (soluble transmitting factors) is not completely understood. However, different studies suggested that calcium fluxes, NO [14], and ROS [15], as well as cytokines including TGF-beta [16,17], IL-8 [18,19], and TNF-alpha [20,21] work as mediators of bystander responses. Furthermore, studies suggest that microvesicles and exosomes are involved in radiation-induced bystander signaling [22,23]. Much like the SASP, cells expressing a radiation-induced BE possess a distinct secretory phenotype [24]. Naïve bystander cells exposed to signals from BE-expressing cells are themselves recruited into the BE-expressing population. In addition to direct irradiation of a progenitor cell population, GI also is inducible through exposure to signals from BE-expressing cells. When triggered this way, GI is considered to be a BE.

### 1.4. Secretion of, and Response to, Molecular Signals

Exosomes, in part, mediate radiation BE signaling [22,23,25]. Exosomes are membrane-bound extracellular vesicles of endocytic origin. They vary in size from approximately 40 to 100 nm (the range is still debated), and are secreted with various contents such as miRNA, protein [26,27], and DNA [28]. The release of these vesicles from BE-expressing cells induces chromosomal aberrations in unirradiated cells through the miRNA and protein, which were shown to work in a synergistic manner [25]. As with the radiation BE, exosomes mediate signals secreted by senescent cells that transfer a stress response to non-senescent cells [29]. Recently, Borghesan et al. reported that exosomes transmit the senescent phenotype to other cells leading to a change in the tissue environment. The results from a combination of different assays such as proteomic analysis, Cre-loxP reporter systems, and RNAi screens demonstrate that exosomes form part of the senescent secretome and mediate paracrine senescence via the activation of a non-canonical interferon (IFN) pathway [30]. These studies have led to the speculation that exosomes could trigger tissue degeneration during ageing and age-related disease [30].

Senescent cell exosomes contain miRNAs shown to regulate senescence-associated gene expression [29]. Some of these changes resemble the stress response associated with tissue trauma. Additionally, and of particular interest here, the list of molecules released into senescent cell cultures overlaps with that of low passage cells undergoing radiation BE. Shared molecular signaling creates the potential for either process to influence, or be influenced by, the other. For example, radiation induces DNA double-strand breaks (DSBs), and this DNA damage initiates cell cycle checkpoints. An identical molecular response is triggered by unprotected chromosome ends produced by progressive telomere shortening [31,32]. Thus, directly irradiated cells and senescent cells activate some of the same pathways. In addition, signals from senescent cells communicate a stress response to non-senescent cells through exosomes similar to the radiation BE [29]. Here, we show that the bystander effects of radiation include the induction of senescence, and that exosomes play a prominent role in the transmission of BE-inducing signals.

## 2. Materials and Methods

### 2.1. Cell Culture and Irradiation

FSF210316B are primary human fibroblasts isolated from neonatal foreskin according to a previously published method [33] conducted under the ethical approval of Oxford Research Ethics Committee Reference 10/H0605/1. The cells were isolated on 21 March 2016. The cells tested negative for mycoplasma. They were authenticated by the internal validation system based on fibroblast-specific isolation procedure followed by visual confirmation of morphology and staining with antibodies against actin, cytokeratin, and CD31 protein, which collectively identifies fibroblasts, keratinocytes, and microvascular endothelial cells. The cells were last tested in May 2019. FSF210316B cells were cultured at 3 × 106 (3 million cells) cells/T175 for 38.5 h in minimal essential media (Dulbecco’s modified Eagle’s Medium-high glucose) supplemented with 10% fetal calf serum and 1% penicillin/streptomycin and routinely subcultured prior to confluence. Cells maintained in a humidified 37 °C incubator with 5% CO_2_. Cells were transported to Oxfords radiation facility in tissue culture flasks. The confluence of the culture prior to irradiation was almost 70%. Cells were subsequently exposed to 0, 0.1, 2, and 10 Gy X-rays before being returned to standard culture conditions described above at Oxford Brookes University.

For experiments, the term “young cells” is applied to the cells that have ~0% senescent cells, passage 2. In comparison, the term “older cells” applies to the cells that have ~17% senescent cells, passage 29.

### 2.2. Population Doublings

Cell growth/proliferation was monitored by determining population doublings (PD). Briefly, 1.5 million cells were seeded into a T75 flask, after 48–72 h (depending on confluence) cells were harvested and counted. Population doublings were determined using the formula PD = c × (log10(f) − log10(i)) + PDi, where (c) is the constant 3.322, f is the final cell count, (i) is the initial number of cells, and (PDi) is the starting number of PD.

### 2.3. Exosome Isolation

Cell media (supernatant) were collected from cells after 1, 6, and 24 h following irradiation. Cell media were centrifuged at 2000× *g* to remove dead cells and apoptotic bodies and any other larger contaminants. The supernatant was then centrifuged at 10,000× *g* for 30 min to clear the sample of microvesicles. This was followed by collection of the supernatant and ultracentrifugion at 120,000× *g* for 1.5 h. The supernatant was discarded and exosome pellet collected in 200 µL of two times 0.22 µm filtered PBS.

### 2.4. Exosome Characterization

Exosome characterization for size and concentration were performed using Izons qnano transient resistive pulse sensing platform as described in [34]. Briefly, exosomes were collected in 200 µL twice filtered PBS. Samples were diluted 1:20 in twice filtered PBS. Samples were run through disposable nanopores for a maximum of 10 min or until 500 exosomes have been counted.

### 2.5. Exosome Transfer for Bystander Experiments (Exosome Bystander)

Exosomes (from each irradiated group) were isolated and collected in 200 µL PBS as above at 24h following irradiation (as the highest yield per flask of exosomes was observed 24 h following irradiation), 10 µL was removed and kept aside for quantification (size and concentration). The remaining exosome sample was added to unirradiated young cell cultures and incubated for 24 h before further analysis.

### 2.6. Media Transfer for Bystander Experiments (Media Bystander)

The irradiated media were collected and filtered at 24 h following irradiation, and then they were added to FSF unirradiated young cells and incubated for 24 h before subjecting to senescence analysis.

The media and exosome transfer experiments were carried out once with 3 technical repeats.

### 2.7. Beta-Gal Senescence Staining

Senescence was performed according to the manufacturer’s instructions. Briefly, 20,000 cells were seeded onto sterilized cover slips in a 6-well plate with 2 mL of media in each well. After 24 h cells were fixed (fixation buffer 10× containing 20% formaldehyde, 2% glutaraldehyde, 70.4 mM Na_2_HPO_4_, 14.7 mM KH_2_PO_4_, 1.37 M NaCl, and 26.8 mM KCl) for 7 min followed by 3 washes with filtered DPBS. Cells were then incubated with freshly made staining solution for a minimum of 18 h at 37 °C without CO_2_. Coverslips were washed and the cells were finally incubated with 70% glycerol in the fridge.

Slides were coded and manually counted in a blind fashion. Cells were counted as senescent if they demonstrated positive β-Gal staining. At least 500 cells were scored per group.

### 2.8. Statistical Analysis

The *p* values of raw data from all experimental groups were compared and calculated. Data were examined for normality. The senescence data was shown not to have normal distribution, thus it was further subjected to Fisher’s exact test to calculate the *p* values. *p* values ≤ 0.05 were considered statistically significant.

## 3. Results

### 3.1. Senescence Induced by Direct Exposure to Radiation

**Figure 1:**
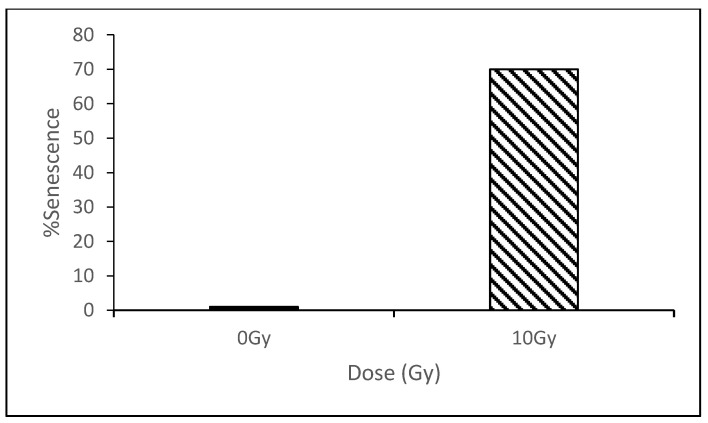
Following exposure to 10 Gy irradiation, 70.9% of cells showed a senescence phenotype compared to <1% for sham exposed cells.

Senescence was initially quantified in response to 10 Gy direct IR exposure. Cells were kept in culture for an additional 5 passages (10 days) and then examined for the induction of senescence as determined by β-galactosidase (β-gal) staining. Less than 1% of the control cells were senescent, this represents a background level of senescence for FSF210316B cells cultured under these conditions. In contrast, 70.9% of the 10 Gy-irradiated cells were senescent demonstrating a high level of senescence induction (Figure 1).

### 3.2. Senescence vs. Dose and Time

**Figure 2:**
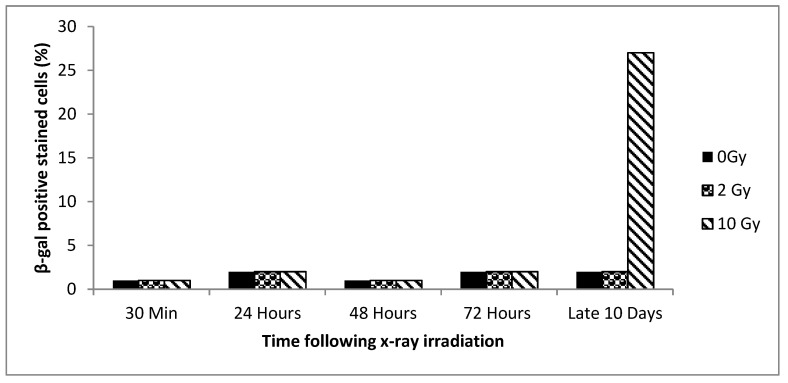
Results for younger cells. The percentage senescence at different time points (30 min, 24 h, 48 h, 72 h, and 10 days) after X-ray irradiation with 2 Gy or 10 Gy is shown. There is a significant difference in senescence in the 10 Gy samples between the 30 min sample and the 10 day sample (*p* value < 0.0001).

Younger cells from cultures with ~0% senescence initially were exposed to 0, 2, or 10 Gy X-rays and tested for senescence induction at 30 min, 24 h, 48 h, 72 h, and 10 day time points. As seen in Figure 2 a low, but nonsignificant, level of senescence was induced by 2 Gy (~2% of the culture) at 10 days, and a much higher level in the 10 Gy/10 day cultures (~27%). Other dose and time points were not statistically significant.

**Figure 3:**
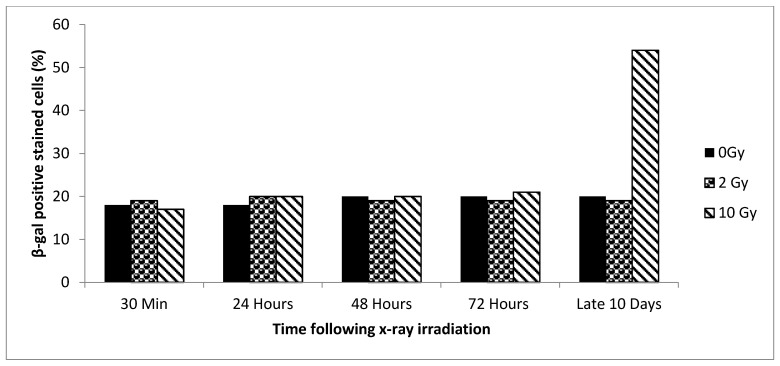
Results for older cells (~17% senescent cells). The percentage senescence over different time points (30 min, 24 h, 48 h, 72 h, and 10 days) after X-ray irradiation with 2 Gy or 10 Gy is shown. The highest level of senescence is found in 10 Gy cells 10 days after radiation. We found a significant difference for all three radiation groups when comparing the 30 min sample to the 10 day samples (0 Gy *p* Value 0.0094, 2 Gy *p* Value 0.0080, and 10 Gy; *p* value < 0.0001).

Repeating the experiment with older cells (~17% senescence initially) resulted in higher levels of senescence. A significant increase in senescence was observed in culture exposed to 10 Gy and examined after 10 days, as shown in Figure 3. As with the younger cells, maximum senescence occurred at 10 days. Approximately 18–20% of the 0 Gy cultures had become senescent as the result of passaging. Therefore, we estimate that of the 54% senescence seen in 10 Gy/10 day cultures, about 35% was induced by radiation. Radiation induced only 26% of the observed senescence in younger cells suggesting that radiation may have a somewhat greater senescence-inducing effect on older cells.

### 3.3. Senescence Bystander Effect

**Figure 4:**
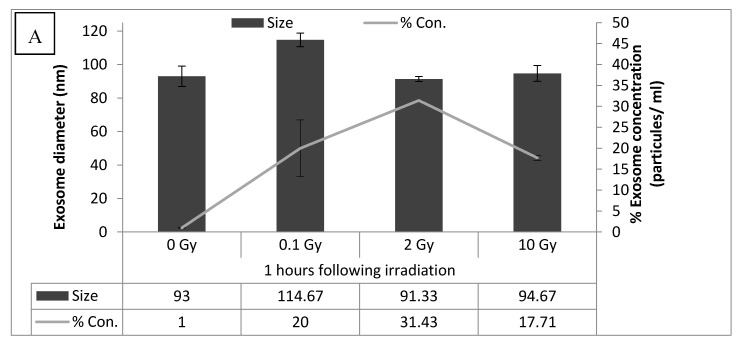

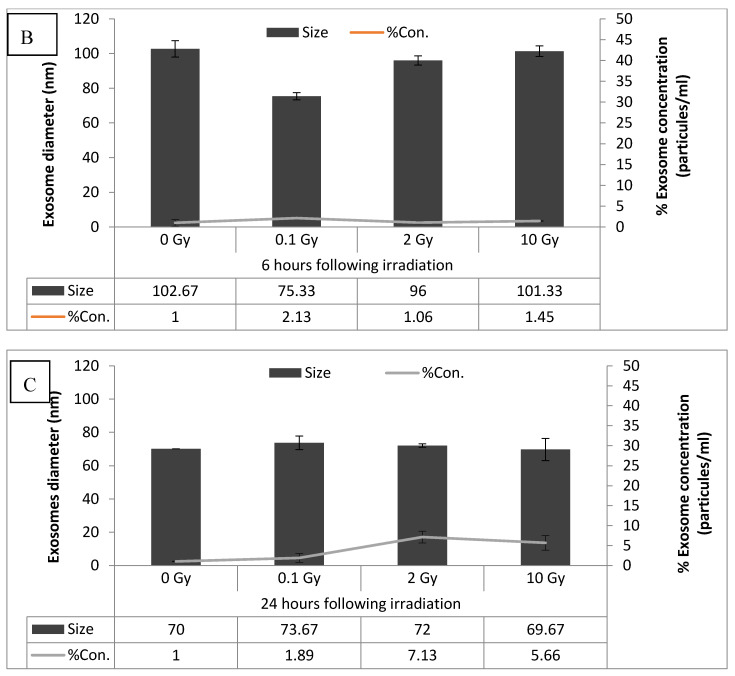
Panels (**A**–**C**) show exosome diameter and the concentration of exosomes at 1, 6, and 24 h after irradiation with doses of 0, 0.1, 2, and 10 Gy. The highest yield of exosomes was observed 24 h following irradiation for all dose points. Therefore, the 24 h time point was chosen for exosome extraction for use in experiments. The exosome concentration data was normalized to the corresponding control for each time endpoint.

After investigating direct induction of senescence through IR we then explored the potential for these effects to be further mediated through bystander signaling. The production and release of exosomes from irradiated cells into culture media was investigated. For these experiments we chose to work exclusively with cells at a passage corresponding to the younger cells investigated above. Medium was collected from cultures at 1, 6, and 24 h after exposure to 0, 0.1, 2, and 10 Gy X-rays. Using tunable resistive pulse analysis, the average size of the exosomes and their concentration were determined. As shown in Figure 4, the size of exosomes was within the expected range (30–150 nm). At every dose the concentration of exosomes was found to increase with post-irradiation time compared to the corresponding controls. This indicates that under our in vitro culture conditions cells release exosomes into the medium at a greater rate than they are removed by endocytosis or other means. Of all exosomes in a culture at any given time point, some were seeded into the culture at the time of passaging and others were released into the medium after irradiation. Of the three time points, the 24 h cultures had the most exosomes, and it also had the largest fraction of post-irradiation exosomes. For these reasons, 24 h was chosen for further experiments.

**Figure 5:**
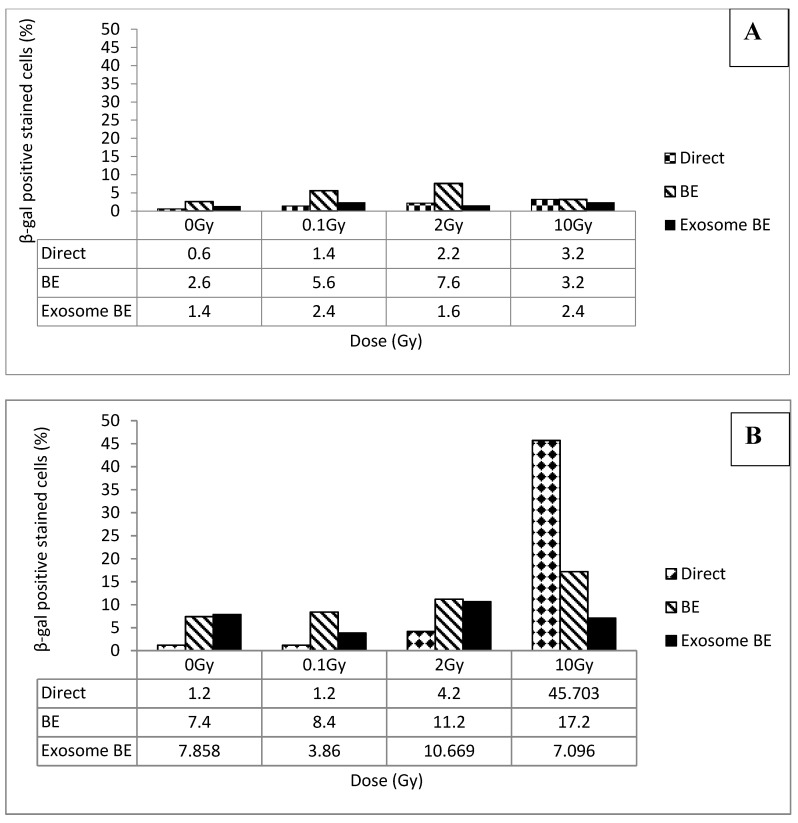
The induction of senescence was analyzed in cells exposed directly to radiation, or to media or exosomes from irradiated cells at 24 h (panel **A**) and 15 days (panel **B**) following irradiation. Panel (**A**) shows an increase in the percentage of cells experiencing senescence following exposure to irradiated media from 0.1 Gy (a significant increase, *p* = 0.0252, at *p* < 0.05) and 2 Gy (a significant increase, *p* = 0.0008, at *p* < 0.05) (media bystander effect) compared to their corresponding control. However, a small increase in cells experiencing senescence was observed at 2 Gy (insignificant increase, *p* = 0.0558, at *p* < 0.05) and 10 Gy (a significant increase, *p* = 0.0041, at *p* < 0.05) direct irradiation. Panel (**B**), shows a significant increase in the level of senescent cells in the direct irradiated 10 Gy group and media bystander 10 Gy group compared to their corresponding controls. Interestingly, cells cultured with exosomes from irradiated cells at 10 Gy showed a reduction in the level of senescence.

As a test for the induction of senescence through a bystander mechanism, cell cultures were exposed directly to radiation, to media from directly irradiated cells, or to exosomes extracted from the media of directly irradiated cultures. Four doses were chosen for the test (0, 0.1, 2, and 10 Gy), and cultures were examined for senescence at four post-treatment times (24 h, 15 days, 30 days, and 45 days). Results shown in Figure 5 demonstrate an increase in the percentage of senescent cells at 24 h following transfer of irradiated media from 0.1 Gy and 2 Gy cultures when compared to the 0 Gy. These results demonstrate the existence of a radiation-activated media transfer senescence bystander effect, and that exosomes are one media component that contains senescence-inducing signals.

**Figure 6:**
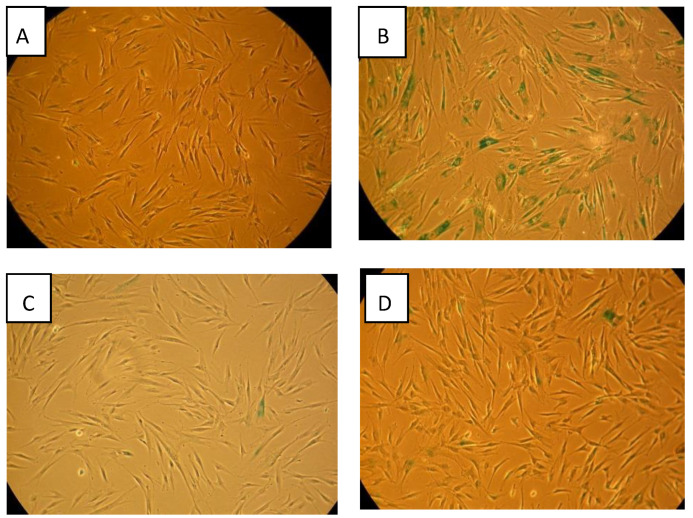
FSF210316B cells, 15 days following X-ray irradiation with senescence-associated beta-galactosidase staining (blue) in 0 Gy control (**A**), 10 Gy x-irradiated cells (**B**), 0.1 Gy (**C**), and 2 Gy (**D**).

As seen in our earlier results, 10 Gy direct irradiation induces substantial senescence (~46%) at five passages (15 days) following exposure as shown in Figure 5, the highest level observed, as shown in Figure 6. The 10 Gy media bystander cultures also had substantial senescence (~17%). Interestingly, all four media bystander cultures displayed elevated senescence in comparison to the unirradiated no-transfer control. Stress associated with media transfer, such as temperature and pH changes, may activate a senescence pathway in some of the cells. Exosome transfer had a similar effect, although in some cases less pronounced, senescence-inducing effect than in media transfer.

**Figure 7:**
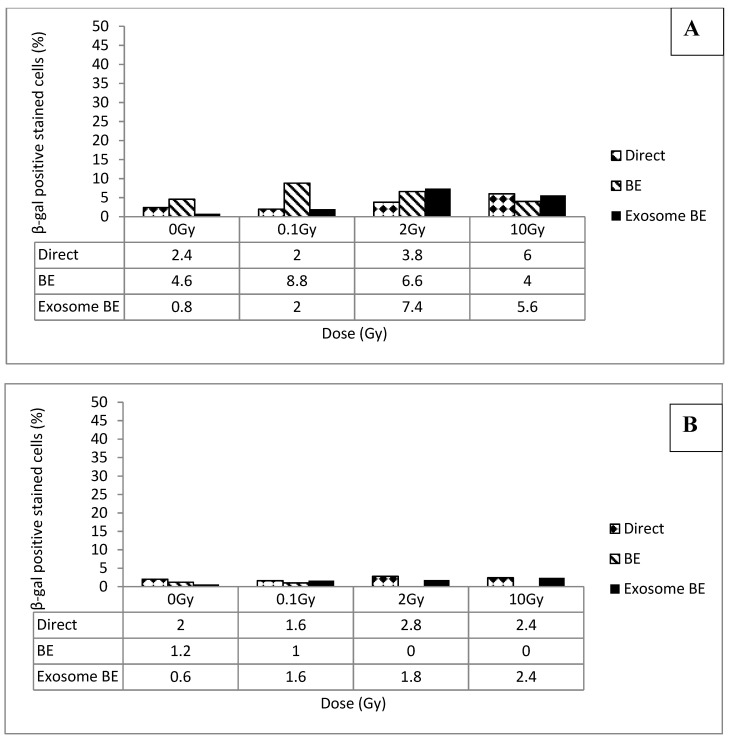
Percentage of senescent cells in control and irradiated FSF210316B cells at 10 passages, (30 days, panel **A**) and 45 days (panel **B**) following irradiation, following irradiation. Panel (**A**), the data show an increase in the level of senescent cells in the 2 Gy (a significant increase, *p* = 0.00001, at *p* < 0.05) and 10 Gy (a significant increase, *p* = 0, at *p* < 0.05) exosome bystander groups, 2 Gy (insignificant, *p* = 0.274) and 10 Gy (a significant increase, *p* = 0.0071, at *p* < 0.05) direct irradiated groups, and the 0.1 Gy (a significant increase, *p* = 0.016, at *p* < 0.05) media bystander group.

By 10 passages (30 days) senescence levels had dropped under most experimental conditions as shown in Figure 7. The drop in senescence levels in directly irradiated cultures might be explained by the dilution of senescent cells with passaging followed by repopulation of cultures with the increase of viable cells. Decreasing bystander senescence is possibly explained for BE persistence by that fact that once naïve/unirradiated cells are exposed to BE-inducing signals, and are recruited into the BE-expressing population, they themselves begin to produce BE-inducing signals. Following cell division in a culture expressing a BE, daughter cells are exposed to these BE-inducing signals from parent cells. Thus, daughter cells are initiated early in their existence and begin producing the bystander signal themselves. The bystander signal remains high as a culture grows and is passed on to new cultures with each passage. Why then is the senescence BE not self-sustaining? We speculate that the senescence BE differs in one important way: Only replicatively competent cells can become parents; therefore, parental cells must be resistant to the senescence-inducing signal. Daughter cells may inherit this trait. If this explanation is correct, both the number of senescent cells and the bystander-inducing signal will be diluted at each passage.

After 15 passages (45 days), senescence levels in cultures initiated from cells directly exposed to radiation, or from cells exposed to media or exosomes from irradiated cells, have dropped to at or near background levels as shown in Figure 6. Thus the senescence media BE and exosome BE appear to be transient phenomena at the cell culture level. However the conversion of a portion of clonogenically competent cells to non-clonogenic senescent cells diminishes a culture’s replicative potential (passages until the culture senesces) and therefore recovery of the culture is not complete.

## 4. Discussion

Our results confirm the existence of a radiation-induced senescence bystander effect. Molecular senescence-inducing signals were present in the media of irradiated cultures and also in exosomes isolated from this media. Under our experimental conditions, the proportions of senescent and non-senescent cells observed at each passage were influenced by multiple dose and time-dependent kinetic processes acting simultaneously. Although experiments were not designed to elucidate these dependencies, we surmise they were influenced by the following processes. First, entry into senescence, or more precisely for our purposes the synthesis of β-gal, does not occur immediately after irradiation. Rather it is delayed to allow development of the senescent phenotype [8] and likely requires cell cycle progression. Second, similarly there may be a lag between triggering of a senescence program by exposure to BE-inducing signals and the synthesis of β-gal in sufficient quantity to mark a cell as senescent. Nelson et al., (2012) found that senescent human fibroblast cells can induce a bystander effect, spreading senescence in intact neighboring fibroblasts in vitro [35]. Third, radiation-induced cell cycle effects, such as the G2 DNA damage block, result in both a depression of growth rate and entry of cells into an observable senescent state. Moreover, factors in irradiated culture medium modulate the G2 block [36]. Fourth, growth rate may be perturbed by radiation, by bystander signals, or by both. Fifth, details of the passaging procedure, which includes dilution of both senescent and non-senescent cells followed by division of only the non-senescent cells to repopulate culture flasks, also affect the proportions of senescent and non-senescent cells observed at each passage. All of these processes, and possibly others, make it difficult to construct simple mathematical expressions for the dose and time dependencies of radiation-induced senescence.

Exosomes were found to convey a senescence-inducing signal, much as they do in the other bystander effects of radiation [12]. However, the experiments do not rule out other means for conveying the signal between cells such as passing through gap junctions, being transported within non-exosomal EV, or by diffusion of unencapsulated molecules after release into the extracellular medium. Borghesan et al. (2019) have found that the soluble fraction and small extracellular vesicles from senescent cells are responsible for mediating paracrine senescence to nearby cells [30].

Induction of bystander senescence by low radiation doses has implications for radiation risk assessment [37]. Accumulation of senescent cells in normal tissue is thought to be a key driver of aging. Radiation-induced senescence, whether through direct irradiation or a bystander mechanism, has the potential to increase the burden of senescent cells and accelerate the aging process. This type of risk has yet to be evaluated. There may also be medical implications of bystander senescence for patients undergoing radiotherapy. These include a beneficial contribution to the tumor sterilizing effect of radiation, or to suppression of replication and division of damaged cells within the tumor or in the tumor margin. However, there is a risk that organ performance may decrease as a result of an increase in senescent cells. Further research will be required to elucidate the mechanisms of bystander senescence as well as its impact on human health.

## Abbreviations

BE	bystander effect
GI	genomic instability

## References

[1] Schafer M.J., White T.A., Iijima K., Haak A.J., Ligresti G., Atkinson E.J., Oberg A.L., Birch J., Salmonowicz H., Zhu Y. (2017). Cellular senescence mediates fibrotic pulmonary disease. Nat. Commun..

[2] Pacheco-Rivera R., Arellanes-Robledo J., García de León M.C., Shibayama M., Serrano-Luna J. (2017). The Role of Senescence in Hepatic Diseases. Liver Pathophysiology: Therapies and Antioxidants.

[3] Lehmann B.D., Paine M.S., Brooks A.M., McCubrey J.A., Renegar R.H., Wang R., Terrian D.M. (2008). Senescence-associated exosome release from human prostate cancer cells. Cancer Res..

[4] Tchkonia T., Zhu Y., van Deursen J., Campisi J., Kirkland J.L. (2013). Cellular senescence and the senescent secretory phenotype: Therapeutic opportunities. J. Clin. Investig..

[5] Childs B.G., Gluscevic M., Baker D.J., Laberge R.M., Marquess D., Dananberg J., van Deursen J.M. (2017). Senescent cells: An emerging target for diseases of ageing. Nat. Rev. Drug Discov..

[6] Martínez-Zamudio R.I., Robinson L., Roux P.F., Bischof O. (2017). SnapShot: Cellular Senescence Pathways. Cell.

[7] Wiley C.D., Liu S., Limbad C., Zawadzka A.M., Beck J., Demaria M., Artwood R., Alimirah F., Lopez-Dominguez J.A., Kuehnemann C. (2019). SILAC Analysis Reveals Increased Secretion of Hemostasis-Related Factors by Senescent Cells. Cell Rep..

[8] Basisty N., Kale A., Jeon O.H., Kuehnemann C., Payne T., Rao C., Holtz A., Shah S., Sharma V., Ferrucci L. (2020). A proteomic atlas of senescence-associated secretomes for aging biomarker development. PLoS Biol..

[9] Sabin R.J., Anderson R.M. (2011). Cellular Senescence—Its role in cancer and the response to ionizing radiation. Genome Integr..

[10] Day R.M., Snow A.L., Panganiban R.A.M. (2014). Radiation-induced accelerated senescence: A fate worse than death?. Cell Cycle.

[11] Morgan W.F. (2012). Non-targeted and Delayed Effects of Exposure to Ionizing Radiation: I. Radiation-Induced Genomic Instability and Bystander Effects In Vitro. Radiat. Res..

[12] Kadhim M., Salomaa S., Wright E., Hildebrandt G., Belyakov O., Prise K., Little M. (2013). Non-targeted effects of ionising radiation-Implications for low dose risk. Mutat. Res..

[13] Kadhim M.A., Hill M.A. (2015). Non-targeted effects of radiation exposure: Recent advances and implications. Radiat. Prot. Dosim..

[14] Shao C., Prise K.M., Folkard M. (2008). Signaling factors for irradiated glioma cells induced bystander responses in fibroblasts. Mutat. Res..

[15] Matsumoto H., Hamada N., Takahashi A., Kobayashi Y., Ohnishi T. (2007). Vanguards of paradigm shift in radiation biology: Radiation-induced adaptive and bystander responses. Radiat. Res..

[16] Burr K.L., Robinson J.I., Rastogi S., Boylan M.T., Coates P.J., Lorimore S.A., Wright E.G. (2011). Radiation-induced delayed bystander-type effects mediated by hemopoietic cells. Radiat. Res..

[17] Iyer R., Lehnert B.E., Svensson R. (2000). Factors underlying the cell growth-related bystander responses to alpha particles. Cancer Res..

[18] Facoetti A., Ballarini F., Cherubini R., Gerardi S., Nano R., Ottolenghi A., Prise K.M., Trott K.R., Zilio C. (2006). Gamma ray-induced bystander effectin tumour glioblastoma cells: A specific study on cell survival, cytokine release and cytokine receptors. Radiat. Prot. Dosim..

[19] Narayanan K., Williamson R., Zhang Y., Stewart A.F., Ioannou P.A. (1999). Efficient and precise engineering of a 200 kb beta-globin human/bacterial artificial chromosome in E. coli DH10B using an inducible homologous recombination system. Gene Ther..

[20] Moore S.R., Marsden S., Macdonald D., Mitchell S., Folkard M., Michael B., Goodhead D.T., Prise K.M., Kadhim M.A. (2005). Genomic instability in human lymphocytes irradiated with individual charged particles: Involvement of tumor necrosis factor alpha in irradiated cells but not bystander cells. Radiat. Res..

[21] Kadhim M.A., Hill M.A., Moore S.R. (2006). Genomic instability and the role of radiation quality. Radiat. Prot. Dosim..

[22] Al-Mayah A.H.J., Irons S.L., Pink R.C., Carter D.R.F., Kadhim M.A. (2012). Possible role of exosomes containing RNA in mediating nontargeted effect of ionizing radiation. Radiat. Res..

[23] Jella K.K., Rani S., O’Driscoll L., McClean B., Byrne H.J., Lyng F.M. (2014). Exosomes are involved in mediating radiation induced bystander signaling in human keratinocyte cells. Radiat. Res..

[24] Calveley V.L., Khan M.A., Yeung I.W., Vandyk J., Hill R.P. (2005). Partial volume rat lung irradiation: Temporal fluctuations of in-field and out-of-field DNA damage and inflammatory cytokines following irradiation. Int. J. Radiat. Biol..

[25] Al-Mayah A., Bright S., Chapman K., Irons S., Luo P., Carter D., Goodwin E., Kadhim M. (2015). The non-targeted effects of radiation are perpetuated by exosomes. Mutat. Res..

[26] Pant S., Hilton H., Burczynski M.E. (2012). The multifaceted exosome: Biogenesis, role in normal and aberrant cellular function, and frontiers for pharmacological and biomarker opportunities. Biochem. Pharmacol..

[27] Record M., Subra C., Silvente-Poirot S., Poirot M. (2011). Exosomes as intercellular signalosomes and pharmacological effectors. Biochem. Pharmacol..

[28] Zhang Y., Liu Y., Liu H., Tang W.H. (2019). Exosomes: Biogenesis, biologic function and clinical potential. Cell Biosci..

[29] Maser R.S., DePinho R.A. (2004). Telomeres and the DNA damage response: Why the fox is guarding the henhouse. DNA Repair.

[30] Borghesan M., Fafián-Labora J., Eleftheriadou O., Carpintero-Fernández P., Paez-Ribes M., Vizcay-Barrena G., Swisa A., Kolodkin-Gal D., Ximénez-Embún P., Lowe R. (2019). Small Extracellular Vesicles Are Key Regulators of Non-Cell Autonomous Intercellular Communication in Senescence via the Interferon Protein IFITM3. Cell Rep..

[31] Slijepcevic P. (2011). Telomere length measurement by Q-FISH. Methods Cell Sci..

[32] Finnon P., Wong H.P., Silver A.R., Slijepcevic P., Bouffler S.D. (2001). Long but dysfunctional telomeres correlate with chromosomal radiosensitivity in a mouse AML cell line. Int. J. Radiat. Biol..

[33] Holliman G., Lowe D., Cohen H., Felton S., Raj K. (2017). Ultraviolet Radiation-Induced Production of Nitric Oxide:A multi-cell and multi-donor analysis. Sci. Rep..

[34] Maas S.L., Broekman M.L., de Vrij J. (2017). Tunable Resistive Pulse Sensing for the Characterization of Extracellular Vesicles. Methods Mol. Biol..

[35] Nelson G., Wordsworth J., Wang C., Jurk D., Lawless C., Martin-Ruiz C., von Zglinicki T. (2012). A senescent cell bystander effect: Senescence-induced senescence. Aging Cell.

[36] Cheong N., Zeng Z.C., Wang Y., Iliakis G. (2001). Evidence for factors modulating radiation-induced G2-delay: Potential application as radioprotectors. Phys. Med..

[37] Squillaro T., Galano G., De Rosa R., Peluso G., Galderisi U. (2018). Concise Review: The Effect of Low-Dose Ionizing Radiation on Stem Cell Biology: A Contribution to Radiation Risk. Stem Cells.

